# Enhancing Non-Small Cell Lung Cancer Survival Prediction through Multi-Omics Integration Using Graph Attention Network

**DOI:** 10.3390/diagnostics14192178

**Published:** 2024-09-29

**Authors:** Murtada K. Elbashir, Abdullah Almotilag, Mahmood A. Mahmood, Mohanad Mohammed

**Affiliations:** 1 Department of Information Systems, College of Computer and Information Sciences, Jouf University, Sakaka 72441, Saudi Arabia; abdull@ju.edu.sa (A.A.); mamahmood@ju.edu.sa (M.A.M.); 2 School of Mathematics, Statistics and Computer Science, University of KwaZulu-Natal, Pietermaritzburg 3209, South Africa; mohammedm1@ukzn.ac.za

**Keywords:** mRNA, miRNA, DNA methylation, multi-omics data, graph attention network

## Abstract

**Background**: Cancer survival prediction is vital in improving patients’ prospects and recommending therapies. Understanding the molecular behavior of cancer can be enhanced through the integration of multi-omics data, including mRNA, miRNA, and DNA methylation data. In light of these multi-omics data, we proposed a graph attention network (GAT) model in this study to predict the survival of non-small cell lung cancer (NSCLC). **Methods**: The different omics data were obtained from The Cancer Genome Atlas (TCGA) and preprocessed and combined into a single dataset using the sample ID. We used the chi-square test to select the most significant features to be used in our model. We used the synthetic minority oversampling technique (SMOTE) to balance the dataset and the concordance index (C-index) to measure the performance of our model on different combinations of omics data. **Results**: Our model demonstrated superior performance, with the highest value of the C-index obtained when we used both mRNA and miRNA data. This demonstrates that the multi-omics approach could be effective in predicting survival. Further pathway analysis conducted with KEGG showed that our GAT model provided high weights to the features that are associated with the viral entry pathways, such as the Epstein–Barr virus and Influenza A pathways, which are involved in lung cancer development. From our findings, it can be observed that the proposed GAT model leads to a significantly improved prediction of survival by exploiting the strengths of multiple omics datasets and the findings from the enriched pathways. Our GAT model outperforms other state-of-the-art methods that are used for NSCLC prediction. **Conclusions**: In this study, we developed a new model for the survival prediction of NSCLC using the GAT based on multi-omics data. Our model showed outstanding predictive values, and the KEGG analysis of the selected significant features showed that they were implicated in pivotal biological processes underlying pathways such as Influenza A and the Epstein–Barr virus infection, which are linked to lung cancer progression.

## 1. Introduction

Cancer survival prediction is crucial to the handling of the patient and the treatment planning. Consequently, multi-omics data and high-throughput technologies give a deep level of detail on the molecular factors that lead to cancer [1]. Multi-omics data come in different forms, making their integration challenging. Nonetheless, the deep learning procedures have the possibility to improve these multi-omics data, thereby improving survival prediction and the determination of such crucial biomarkers of cancer [2]. Multi-omics data consist of multiple layers of biological measurements, such as mRNA, miRNA, or DNA methylation, which offer unique perspectives into the development of cancer [3]. For instance, genomics employs DNA sequencing in order to identify mutations, copy number variations, or any other genetic change [4]. Transcriptomics identifies differential splicing and quantification of the relative levels of gene expression using RNA sequencing [5]. Proteomics estimates the amount and alteration of proteins through mass spectrometry, while metabolomics offers a snapshot of the metabolic state of cells [6]. As for obtaining another prospect of gene regulation, epigenomics also considers changes in the histone structure and DNA methylation [7].

Deep learning involves complex, non-linear interactions and neural networks, which are well suited to incorporating multi-omics data for survival analysis. Several deep learning architectures have been employed for cancer survival prediction. The multi-omics data can be modeled via auto encoders that are used in dimensionality reduction and feature learning [2]. A concatenation autoencoder architecture was used with the multi-omics data to analyze the survival of breast cancer patients [8,9]. Time series omics data are well accommodated by recurrent neural nets, including recurrent neural nets with long short-term memory, or LSTM [10]. Graph convolutional networks are particularly suitable for biological networks, as they leverage the relational structure of data [11]. Some aspects which are critical for the model across different layers of omics can be brought into focus by using attention processes, thereby enhancing the interpretability of the model.

Several recent works have utilized the deep learning techniques to improve the prognosis of lung cancer, integrating multiple orthogonal data from omics profiles. For example, Wang et al. [12] employed attention-based deep learning features to predict the survival of lung cancer patients and determine the important multi-omics biomarkers. The attention mechanism made the model more selective, and by paying attention to some parts of the input data, it was better at predicting survival. In another important study, Jacob G. Ellen [13] investigated the NSCLC survival prediction using a composite of multi-omics data classes that comprised microRNA, mRNA, DNA methylation, lncRNA, and clinical data. Using denoising autoencoders to compress and integrate these data modalities, the authors identified that the lncRNA–clinical data fusion model had the highest concordance indices for survival prediction for LUAD and LUSC, with concordance indices (C-index) of 0.69 and 0.62, respectively. Zhang, J [14] also used data from the SEER and adapted a nomogram model to estimate the performances of NSCLC patients. Based on the clinical features of these patients, clinically related factors were selected and a nomogram for screening Overall Survival (OS) was established which had a C-index of 0.714, which was quite accurate as a means of prediction. At the same time, She Y et al. [15] proposed the DeepSurv, which is a deep learning survival neural network for NSCLC. This model was then compared with other approaches and was found to be superior, with a C-index of 0.739. Their research also showed that the patients who adhered to the treatment plans derived from the DeepSurv model enjoyed higher survival rates. Furthermore, Zhang D et al. [16] built an interpretable deep learning model that was applied to small cell lung cancer (SCLC). There were numerous clinical features that were associated with the survival analysis, such as age, sex, the primary tumor site, the TNM stage, tumor size, surgery in the recent tumor, chemotherapy, radiotherapy, and a history of malignancy. These features were incorporated into the development of a tool that enabled the creation of survival curves by inputting clinical data automatically, which provided fairly accurate survival predictions and real value for clinicians. Suniy Zheng et al. [17] developed a hybrid deep learning model using clinical variables (age and clinical stage) and image features extracted from pretreatment CT for predicting 2 years of survival in NSCLC patients. The model was trained on 189 patients and validated on other datasets and was found to have a median AUC of 0.76 on the test set of UMCG and 0.64 on the Maastro test set. It was able to differentiate between high- and low-mortality-risk patients, which opens up a possibility for use in clinical decision making. Yimeng Guo et al. [18] built an NSCLC survival prediction model with 523 participants. Three methods were used for variable selection: univariate Cox regression, LASSO regression, and random survival forest (RSF). Multivariate Cox regression was used for building the final predictive model. Using external validation datasets, the LASSO regression method with an N-stage neutrophil–lymphocyte ratio (NLR) and first-line treatment appeared to be the best model in the validation. Recent studies have shown that deep learning and multi-omics data integration are valuable elements in directing improved lung cancer survival. Modern techniques like attention mechanisms, autoencoders, or deep survival networks allowed the enhancement of predictive modeling and, consequently, better individualized therapy in the context of the lung cancer treatment trajectory.

While these results are promising, bringing together multi-omics data to discover predictive signatures would also introduce several great challenges for cancer prognosis. Firstly, omics datasets are often heterogeneous in their different scales (e.g., mass-spectrometry intensity levels vary by more than a magnitude of 10^4^), noise levels, and distributions [13]. Furthermore, there is a scarcity of high-quality, large-scale multi-omics datasets that are accompanied by clinical outcomes [19]. This means that future works need to give attention to making their models more interpretable and/or conducting studies with a better data preprocessing approach, as well as to providing publicly available multi-omics datasets. Ensemble deep learning with traditional statistical models [15] is also used to obtain the advantages of both robustness and interpretability. Nevertheless, the deep learning models have the capability to predict the survival rates and to improve the predictors of cancer based on multi-omics data. It is therefore possible for researchers to harness the benefits of the different neural networks’ architectures and apply different biological data types to study cancer survival. Progress in this domain will most certainly have an increase in patients’ survival rates and a more individualized approach to cancer therapy [16,20,21].

The primary motivation for this study stems from the need to improve survival predictions for non-small cell lung cancer (NSCLC) patients, as current methods offer limited accuracy despite advancements in treatment. We propose a novel method for improving the NSCLC survival prediction using graph attention networks (GATs) based on multi-omics data and clinical information. GATs are highly effective for cancer survival prediction because they leverage attention mechanisms to focus on the most biologically relevant features, improving interpretability and predictive accuracy. Additionally, GATs handle the complex relational structure of multi-omics data, allowing the seamless integration of diverse data types, such as mRNA, miRNA, and DNA methylation; this enhances model robustness and performance in high-dimensional, sparse datasets. An integrated multi-omics dataset was developed with mRNA, miRNA, and DNA methylation combined with clinical data to create a robust predictive tool for NSCLC survival in order to provide a better understanding of lung cancer development. To fully model the relationships between the different features of molecules, we utilized the correlation between samples to determine the dependencies. Also, an advanced feature selection through the chi-square test for selecting the features with greater relevance to the survival prognosis was used with the SMOTE, which balances the data to improve the model’s predictive power and generalization. This is evident in the series of experiments that were conducted to ensure comparison, and it shows that the proposed GAT-based model performs better than the other methods when it comes to the survival rate of NSCLC cancer patients. Furthermore, the pathway analysis shows that the most important features that are selected based on the attention weight are biologically significant features linked to cancer-related processes, such as the Influenza A and Epstein–Barr virus infection signaling pathways. This work can advance the field by offering clinicians a more accurate, interpretable model, fostering personalized treatment strategies and supporting the future of precision medicine for NSCLC.

## 2. Materials and Methods

**Figure 1:**
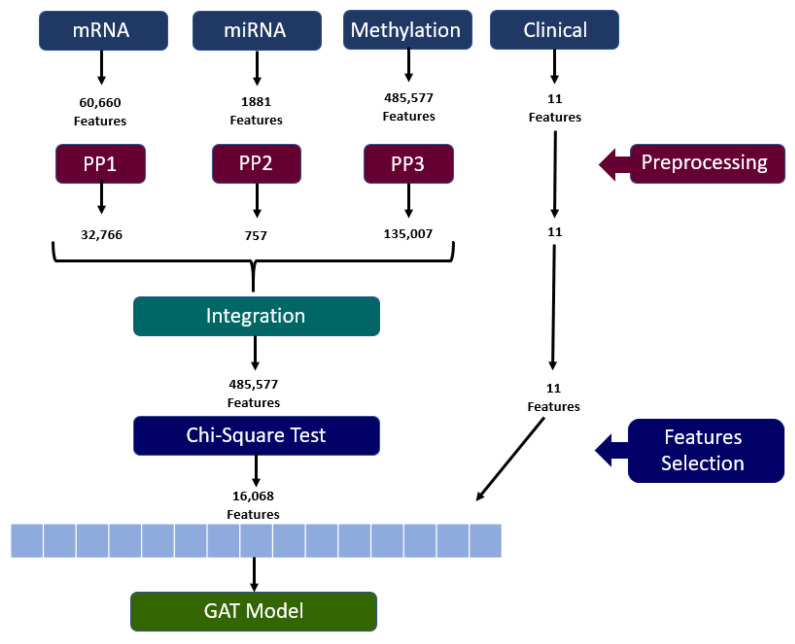
Pipeline for NSCLC survival prediction using GAT.

The complete pipeline of our data analysis approach is depicted in Figure 1 It starts with four types of data: three types of omics data (mRNA, microRNA, and methylation) and clinical information; these are represented by certain numbers of initial features. The data undergo preprocessing, reducing the feature count for each omics data type: the mRNA features are decreased from 60,660 to 32,766; the miRNA features are decreased from 1881 to 757, whereas the methylation features are decreased from 485,577 to 135,007. The clinical information data, which contain 11 features, are analyzed independently. Finally, the three omics data types are merged, and its final set of 168,530 features summarizes all the omics integrations. Analysis is performed to decide on which features to keep; then, feature dependency analysis is conducted using a chi-square test; the best 16,068 features are selected and taken to the GAT model. The steps in the data processing, feature selection, and model building are clearly illustrated by Figure 1, which shows the manner in which the various data types have been incorporated to enhance the NSCLC survival prediction accuracy.

### 2.1. The Omics Data

**Figure 2:**
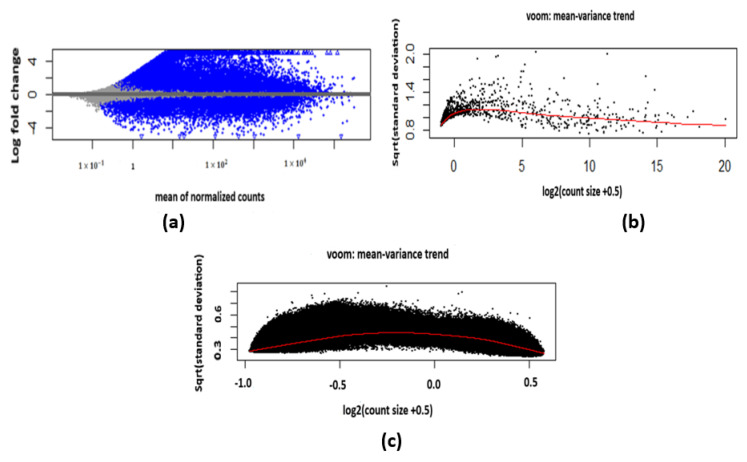
The omics dataset visualization: (**a**) mRNA, (**b**) miRNA, (**c**) DNA methylation.

The GDCquery function [22] is used to create a query for obtaining multi-omics data, which include gene expression quantification, miRNA expression quantification, and DNA methylation data from the GDC portal. This query specifies the data from two TCGA projects using the identifiers TCGA-LUAD (lung adenocarcinoma) and TCGA-LUSC (lung squamous cell carcinoma). This should include both primary tumor samples and solid tissue normal samples so that we can compare the methylation patterns between the two types of tissues and obtain an understanding of the epigenetic changes which accompany lung cancer. The obtained data were preprocessed using the GDCprepare function, which is part of the TCGAbiolinks R package designed for handling TCGA data. Also, the function includes an option which helps in the handling of the downloaded data files from the GDC portal to change the data from a raw form to a format amenable to analysis in R by carrying out preprocesses of the data, such as normalization, filtering, and annotation of the data, to check for consistency of the data received from the GDC portal. In addition, it includes molecular profile data (for example, gene expression, miRNA expression, DNA methylation); thus, comprehensive integrative analyses can be conducted. We used the DESeq2 function version 1.40.2 [23] to analyze gene expression differences by fitting a generalized linear model to compare tumor and normal tissues. The result is presented in the form of count values, and we selected the data so as to remove genes that have low intensity or low variation using the rowSums function. The genes are retained only when the total counts in all the samples exceed the counts of 2 and when there are not less than 4 samples with more than the count of 2. The miRNA expression and DNA methylation data were processed using the limma package version 3.56.2 [24] in R, which aims at the linear modeling of microarray and RNASeq or mRNA data. The package fits a linear model to each miRNA in the expression or to the DNA methylation data to model the association between the expression levels and the experimental conditions (tumor versus normal tissues). With this approach, it is possible to obtain a list of genes or features which have significant differences between the tumor and normal samples; therefore, the non-significant features are filtered out. The visualization of the mRNA data after the differential expression analysis is depicted in Figure 2a. The plot indicates the correlation between the mean of the counts, after data normalization (*x*-axis), with the log fold change (*y*-axis). The diagnostic plots from the voom function of the limma package for miRNA and DNA methylation are presented in Figure 2b,c. These plots reflect the mean variance plot of the data after the voom transformation for miRNA or DNA methylation data; the voom transformation helps to reduce the variability of the data.

### 2.2. The Clinical Information Data

Table 1 presents a summary of the clinical features of the 624 patients of the study group, stressing the demographic and clinical features. Regarding gender distribution, the number of males is 370 (59.29%), while the number of females is 254 (40.71%), and there are no cases of missing data. The patients’ ages vary from 39 to 88 years; the average age is 66.19 years, and the standard deviation is 9.32. A history of previous cancer is noted in 86 patients, which is 13.8% of the patients; in addition, there is one patient with no information reported. Synchronous malignancy is noted in only 2.24% of the patients, where 43 patients’ details were not recorded. The majority of patients (99.52%) had not undergone treatment. The two most common forms of cancer are adenocarcinoma with a prevalence rate of 55.77% and squamous cell carcinoma with a prevalence of 44.23%. Staging of the tumor reveals that 33.49% of the patients are in stages T1 T1a T1b; 51.12% are in stages T2, T2a, T2b; 12.50% are in stage T3; 2.40% are in stage T4; and 0.48% are in stage TX. Furthermore, lymph node staging shows that 69.50% of the patients are in stage N0; 20.38% are in stage N1; 8.19% are in stage N2; 1.77 are in stage N3; and 0.16% are in stage NX. The metastasis stage shows that 69.03% of the patients are in stage M0; 1.93% are in stages M1, M1a, M1b; and 29.03 are in stage MX. The tumor location data are complete, and the most frequent location of the observed tumor was in the upper lobe of the lung, which was found in 57.85% of the cases; this was followed by the lower lobe of the lung, where it was detected in 34.29% of the cases, followed by middle lobe (3.37%), lung NOS (2.24%), overlapping lesions (1.28%), and main bronchus (0.96%). The average of the smoking history, defined in pack-years, is 47.09, and the standard deviation is 28.43. The number of the missing data in the smoking history is 154. 

### 2.3. Chi-Square Test for Feature Selection

We used the chi-square test [25] for feature selection to evaluate and determine the features that are significantly associated with the event “Dead or Alive”. The dataset was simply a set of various types of features or omics data (mRNA, miRNA, and methylation). The chi-square analysis was conducted on the features that were binarized based on the median value. In each of the features, we built a contingency table that represented the association of the binary features with the target variable. We then applied the chi-square test to select the most significant features. The chi-square statistic (*χ*^2^) can be calculated using the formula:

(1) $$\chi^{2}=\sum \frac{{\left(O_{i}-E_{i}\right)}^{2}}{E_{i}}$$

Here, $O_{i}$ refers to the observed frequency for the ith category, and $E_{i}$ is the expected value for the ith category, calculated under the assumption of independence. The expected value $E_{i}$ can be calculated as:

(2) $$E_{i}=\frac{\left(R_{i}\times C_{i}\right)}{N}$$

where $R_{i}$ is the sum of the ith row, $C_{i}$ is the sum of the ith column, and N is the total number of observations. The degrees of freedom for the chi-square test can be calculated using the formula:

(3) $$df=\left(r-1\right)(c-1)$$

where r and c are the number of rows and columns, respectively, in the contingency table.

### 2.4. Kaplan–Meier Survival Curves

The Kaplan–Meier estimator, also known as the product limit estimate, is a non-parametric statistic that estimates the survival function from lifetime data [26]. In medical research, it is commonly used to estimate the proportion of patients surviving to a specified number of years. The Kaplan–Meier curve is a step function that displays the profile of survivals and gives the assembler probability of survival in times. The Kaplan–Meier estimator for the survival function is given by [27]:

(4) $$S\left(t\right)=\prod_{t_{i}\leq t}\left(1-\frac{d_{i}}{n_{i}}\right)$$

where $t_{i}$ is the time of the event, $d_{i}$ is the number of events at time $t_{i}$, and $n_{i}$ represents the number of people at risk ‘just before’ time $t_{i}$. The product runs over all event times up to the time of interest. Censoring happens when a patient refuses to be further included in the study, or is unreachable for follow-up, or when the study ends before the event can be observed [28]. In such cases, the value of the survival time is not known precisely. The Kaplan–Meier estimator deals with censoring by using a method of adjusting the number of persons at risk at each event time, in contrast to what is believed when the censored patients are deemed to have undergone the event. The log-rank test can be used to assess whether there is a significant difference between two or more groups, and the formula to calculate the log-rank test is as follows [28]:

(5) $$\chi^{2}=\frac{{\left[\sum_{i}\left(O_{i1}-E_{i1}\right)\right]}^{2}}{\sum_{i}V_{i1}}$$

where $O_{i1}$ is the observed number of events in group 1 at time $t_{i}$; $E_{i1}$ is the expected number of events in group 1 at time $t_{i}$, calculated according to the null hypothesis; and $V_{i1}$ is the variance of the number of events in group 1 at time $t_{i}$.

### 2.5. The Cox Proportional Hazards (Cox PH)

The Cox proportional hazards (Cox PH) model is one of the most popular statistical models; it is used when analyzing the survival data in order to identify the connection between people’s life durations and one or more predictive variables [29]. The hazard function is assumed to be a product of a baseline hazard that is a function of time and a covariate function that remains constant with time. This is captured in the following key equation of the model:

(6) $$h\left(t|X\right)=h_{0}\left(t\right)exp(X\beta )$$

where $h\left(t|X\right)$ is the hazard which represents the conditional probability of failure at time t given the covariates X; $h_{0}\left(t\right)$ is the hazard at time t when there are no covariates; and exp⁡(Xβ) is the effect of the covariates on the hazard. The model is semi-parametric and hence does not require the specification of the baseline hazard function, and the covariate coefficients are estimated using a method called partial likelihood estimation. As the coefficients of the covariates, the hazard ratios provide further information on the impact concerning the hazard with regard to the covariates. The Cox PH model is widely used in many fields, such as in medical research, especially in disease outcome studies, engineering, and economics. Its assumptions include the parallelism of risks over time, the linearity of covariates, and non-informative censoring, where the pattern for censoring is unrelated to the survival pattern.

### 2.6. Graph Attention Network (GAT)

The graph attention network (GAT) is one of the most advanced architectures in the field of GNNs. It can work with graph structured data using the attention mechanism, which can define the ‘weight’ of the neighboring nodes during training [30,31]. The GAT has improved the efficiency of models that are based on graph structure; therefore, it can capture relationships in the data. Therefore, the GAT can be easily applied to the modeling of bioinformatics problems, such as survival analysis, and can deal with complex data, such as multi-omics data. It can focus on the most important part of the graph structure using the attention coefficients. The attention coefficients are responsible for determining the most important neighboring nodes. The important components of the GAT model are the node features, the attention mechanism, and the aggregation function.

#### 2.6.1. Node Features

Consider a graph G=(V,E) with N nodes, where V is the set of nodes and E is the set of edges. Each node $i\in V_{i}$ is associated with a feature vector $h^{i}\in R^{F}$, where F is the number of features.

#### 2.6.2. Attention Mechanism

The attention mechanism in the GAT computes attention coefficients $e_{ij}$ for each pair of connected nodes (i,j)∈E. These coefficients indicate the importance of node $j^{,s}$ features to node i. The coefficients are computed as follows:

(7) $$e_{ij}=LeakyReLU(a^{T}\left[{Wh}_{i}||{Wh}_{j}\right])$$

where ${W\in R}^{F^{\prime }\times F}$ is a weight matrix, ${a\in R}^{2F^{\prime }}$ is a learnable vector, [⋅ ∣∣ ⋅] denotes concatenation, and LeakyReLU is a non-linear activation function.

#### 2.6.3. Normalization

The attention coefficients are normalized across all neighboring nodes of node i using the softmax function:

(8) $$\alpha_{ij}={softmax}_{j}\left(e_{ij}\right)=\frac{\exp(e_{ij})}{\sum_{k\in N_{i}}\exp(e_{ik})}$$

where $N_{i}$ denotes the set of neighbors of node i.

#### 2.6.4. Aggregation

The normalized attention coefficients $\alpha_{ij}$ are used to compute a weighted sum of the neighboring node features, which updates the feature representation of node i.

(9) $$h_{i}^{\prime }=\sigma \left(\sum_{j\in N_{i}}\alpha_{ij}{Wh}_{j})\right)$$

#### 2.6.5. Multi-Head Attention

To stabilize the learning process and improve model capacity, the GATs employ multi-head attention. Multiple independent attention mechanisms, or heads, are applied, and their outputs are concatenated or averaged:

(10) $$h_{i}^{\prime }=\sigma \left(\frac{1}{K}\sum_{k=1}^{K}\sum_{j\in N_{i}}\alpha_{ij}^{k}W^{k}h_{j}\right)$$

### 2.7. Synthetic Minority Over-Sampling Technique (SMOTE)

The SMOTE generates synthetic samples for the minority class, which is considerably underrepresented in relation to the class that is the majority based on the oversampling approach. By taking the difference between a sample and its nearest neighbor, the SMOTE can generate a new synthetic example in feature space by adding the difference to the sample and multiplying the result by a random number between 0 and 1. It then continues by adding the next nearest neighbor up to a user-defined number. This way the SMOTE can handle the problem of overfitting, which can result from the duplication of the samples of the minority class. Considering that we have minority examples $x_{i}$ and that one of its k-nearest neighbors is $x_{i,nn}$, the equation that is used by the SMOTE to generate a synthetic sample $x_{syn}$ is as follows:

(11) $$x_{syn}=x_{i}+ʎ\times (x_{i,nn}-x_{i})$$

where ʎ is a random number between 0 and 1. This way the SMOTE creates samples that are similar but not identical to the minority class.

## 3. Experimental Setup

We first obtained the RNASeq or mRNA, miRNA, and DNA methylation data for the non-small cell lung cancer (NSCLC) (lung adenocarcinoma (LUAD) and lung squamous cell carcinoma (LUSC)) from the TCGA repository. The downloaded mRNA data were then preprocessed using the DESeq2 package version 1.40.2, which filters out the genes or the features with low counts by using the negative binomial distribution to test for deferential expression. For the preprocessing of the miRNA and DNA methylation data, we used the limma package, which is very effective for expression data that are of a large scale. Limma fits a linear model and utilizes an experimental design that is very complex to test hypotheses, through which it can filter out low-expressed miRNA or low-quality probes for DNA methylation. The three data types are merged based on the sample ID to form multi-omics data. Both mRNA and miRNA are types of RNA molecules that can exhibit high or low expression levels, depending on their biological activity in the cell. On the other hand, DNA methylation is an epigenetic modification where methyl groups are added to cytosine bases, particularly at CpG sites. High methylation at CpG sites typically represses gene expression, while low methylation is associated with increased gene activity, influencing gene regulation. To select the most significant features from the multi-omics data that influence the survival analysis, we used the chi-square test. We implemented a loop that iterates through each feature in the omics dataset and creates a contingency table by binarizing the feature values based on whether they are greater than the feature’s median. In the context of mRNA and miRNA data, the median refers to the middle value of expression levels for a particular gene across all samples, while in DNA methylation data, the median refers to the middle value of the methylation levels at a specific CpG site across all samples. This binarization is necessary because the chi-square test is designed for categorical data. The chi-square test uses the hypothesis testing and determines the features that are associated with the event using the *p*-value. The *p*-value threshold was set to 0.05, indicating that any features that had a *p*-value of less than 0.05 were considered statistically significant and would be selected among the most significant features. This threshold is commonly used in statistical analysis to ensure that the selected features have a high likelihood of being truly associated with the outcome rather than by random chance. The clinical and demographic information data are merged with the most significant features from the multi-omics data. The resulting feature matrix is then combined with the graph that has resulted from the correlation between the samples, where the nodes correspond to the samples and the edges to the known correlation between them; these are then used for training our GAT model. A threshold of 0.7 is used to determine whether the samples are correlated or not.

Before we fed the data to the GAT model, we used the SMOTE, where we set the k_neighbors and the random state parameters to 1 and 42, respectively. The ability of the SMOTE in balancing the dataset that was used for training helped to make the model generalize more. We tried different data combinations in training our model, where the input went through two convolutional layers. The convolutional layers were optimized using the grid search approaches, which try different parameter combinations to tune the model and select the best parameters that are to be used in the model construction. During the course of tuning, our model performance was assessed using the concordance index (C-index) to check the quality of the model across different hyperparameters, and the best was picked based on the C-index obtained during training. The optimized hyperparameters were hidden_dim, num_heads, learning rate (lr), and weight decay. The selected values for these parameters through the grid search approach were as follows: hidden_dim of 64, num_heads of 4, learning rate (lr) of 0.001, and weight decay of 1 × 10^−5^. We implemented 10-fold cross-validation to ensure the model’s reliability and robustness, using the concordance index (C-index) to evaluate model performance across different hyperparameters, selecting the best model based on its C-index during training. The model contained a batch normalization layer after the first GAT layer and a dropout of 0.6 to prevent overfitting. Censored survival data were dealt with using a Cox partial likelihood loss function, and gradient clipping was used to prevent gradients from exploding while the model was being trained. The Cox partial likelihood loss function is a distinct type of loss function for use in survival analysis and is mostly used with the Cox PH model. It is intended for a censored data situation, which is typical for survival analysis, in which the time for the occurrence of an event is not recorded for all participants. We used five-fold cross-validation in our model training to ensure the reliability of the model and to determine the survival outcomes of lung cancer patients.

## 4. Results

Table 2 shows the number of selected features using the chi-square test for each of the omics data types. Also, the table shows the minimum and maximum *p*-value for each omics data type. For the mRNA data, the number of selected features is 2945, where the number of the original features that are passed to the chi-square test is 32,766; the minimum *p*-value for the features is 3.7131 × 10^−7^, where the minimum *p*-value is 0.04998. The table shows that for the miRNA data, the chi-square test selected 77 features out of 757 features, with minimum and maximum *p*-values of 0.0002 and 0.0315, respectively. For the DNA methylation data, the number of selected features is 13,046 out of 135,007, with minimum and maximum *p*-values of 6.9051 × 10^−6^ and 0.0315, respectively; the maximum *p*-values for the selected features for the three omics data types are less than 0.05, which means that all of the features are significant based on our determined threshold, and this indicates that all of the selected features are closely related to the event of the surveillance; therefore, they can be used in building our GAT model.

### 4.1. Exploratory Analysis by Evaluating the Survival Differences between High-Risk and Low-Risk Groups

**Figure 3:**
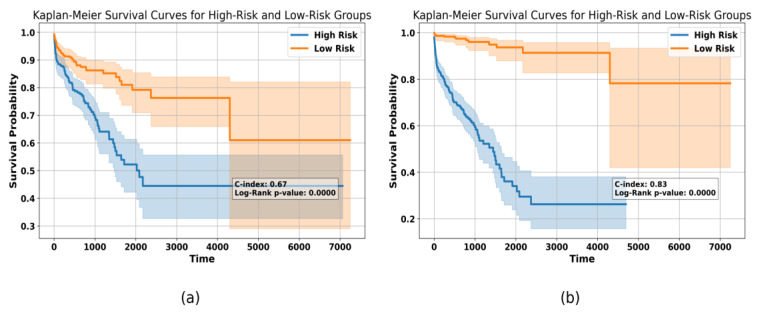
Kaplan–Meier survival curves (**a**) using all of the features and (**b**) using the significant features that were selected using chi-square test.

Figure 3 depicts the results of applying Kaplan–Meier survival curves to our data before and after features selection. We calculated the low- and high-risk groups based on a risk score derived from the features, using the median risk score as a threshold to differentiate between these groups. To calculate the risk score, we first applied principal components analysis (PCA) to the features (mRNA, miRNA, and DNA methylation) to reduce the dimensionality. Then, we dropped the highly correlated features and used chi-square in order to determine the features that were most useful in predicting the patients’ survival. We used Cox proportional hazards together with L2 regularization to obtain the risk score. The survival curves comparison between the high-risk and low-risk patients before applying the chi-square test as a features selection process showed a C-index of 0.67. Nevertheless, after performing the chi-square feature selection, the Kaplan–Meier analysis was improved and obtained a C-index of 0.83. This improvement demonstrates that the selected features are more informative and can better distinguish between the survival outcomes. As these features are more biologically relevant, the Kaplan–Meier curves imply a better and appropriate stratification of the patients, enabling the creation of a better and easily interpretable deep learning model for predicting survival. It increases not only the model’s predictive ability but also the possibility of clinical application by identifying features most relevant to patient outcomes.

Table 3 shows the C-index that is obtained using the five-fold cross validation approach for the different combinations of the omics and the clinical information data. The C-index evaluates the model’s predictive ability in terms of survival. The C-index value that is calculated for the different combinations of the data is between 0.62480 and 0.86322. This range provides information about the significance of the combinations of data in terms of the potential impact on the survival prediction. The highest C-index that was obtained was 0.86322. This value was obtained when combining the mRNA and miRNA data for survival analysis, which means that these two data types together make the survival prediction analysis more reliable. The mRNA and clinical information showed a C-index of 0.86056, which was close to the value that was obtained when combining mRNA and miRNA. On the other hand, the combination of the mRNA, miRNA, and clinical information showed a C-index of 0.86092. We notice that the common data type in these three different combinations is mRNA, and this suggests that mRNA is a very important feature in predicting NSCLC survival. The table also shows that the clinical information data alone have a minimum C-index, which points out the importance of the omics data especially those mRNA and miRNA. However, when integrating the miRNA and DNA methylation data, the C-index value was 0.79529, and with the integration of mRNA and DNA methylation, the value of the C-index was 0.81750; this confirms the importance of the mRNA data. It is also worth mentioning that, regarding the diagnostic values, the DNA methylation that had 13,046 significant features had the C-index of 0.79091, which was lower than that obtained when using either mRNA (0.85676) or miRNA (0.83605). Thus, although the DNA methylation data offer useful information, the separate consideration of methylation data might not immediately predict survival as robustly.

**Figure 4:**
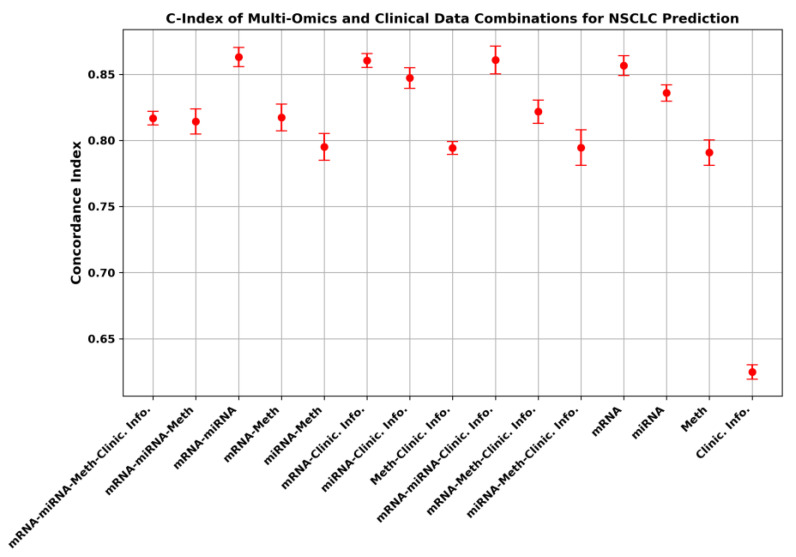
Predictive performance of GAT-based method using different feature combinations.

The concordance index of the different combinations of the molecular data types used in the survival analysis-based prediction of lung cancer is summarized in Figure 4. Figure 4 shows error bars, which are graphical depictions of the data variability that are used to show inaccuracy or uncertainty in reported measurements on graphs. They provide a broad indication of the accuracy of a measurement or, on the other hand, how far the genuine (error-free) value may deviate from the reported value. The type of data combination is represented on the *x*-axis, and the C-index in the *y*-axis. The error bars indicate the standard deviations of the C-index measures, which allow the understanding of the variability and fluctuation of each intended measurement. The C-index of the combinations of these data are represented by single points with red markers, and the standard deviation is presented by the bars. The figure shows that the combination of mRNA, miRNA, DNA methylation, and clinical information data shows a very low error level in the reported C-index, while the combination of miRNA, DNA methylation, and clinical information shows a comparatively large error. The clinical information data, however, have the lowest C-index value, but its error is small.

### 4.2. Comparative Analysis of Predictive Models for Non-Small Cell Lung Cancer

Table 4 provides a comparison of several NSCLC predictive models, which use the C-index as a performance measure. These models vary in their approach and the features used, some of which are multi-omics integrated and clinical data, while others are only clinical data. Our graph attention network approach, which integrates multi-omics data with clinical features, has the highest C-index of 0.85, which is higher than random and thus suggests better predictive ability. On the other hand, Jacob G. Ellen’s method [13], which uses autoencoders with multi-omics data and clinical information, has C-index values of 0.69 and 0.62 for LUAD and LUSC, respectively. The models that are based purely on clinical information data are those used by Zhang, J [14] and She Y et al. [15]. These models obtained C-index values of 0.71 and 0.74 respectively. It is important to note that these models present fairly good predictive ability and are on a par with other models, which, however, utilize multi-omics data. In general, integrating multi-omics data with clinical data about patients with NSCLC, as was the case with our graph attention network approach, enriches the prognostic potential for survival and underscores the utility of multivariate information in creating accurate predictive models.

**Figure 5:**
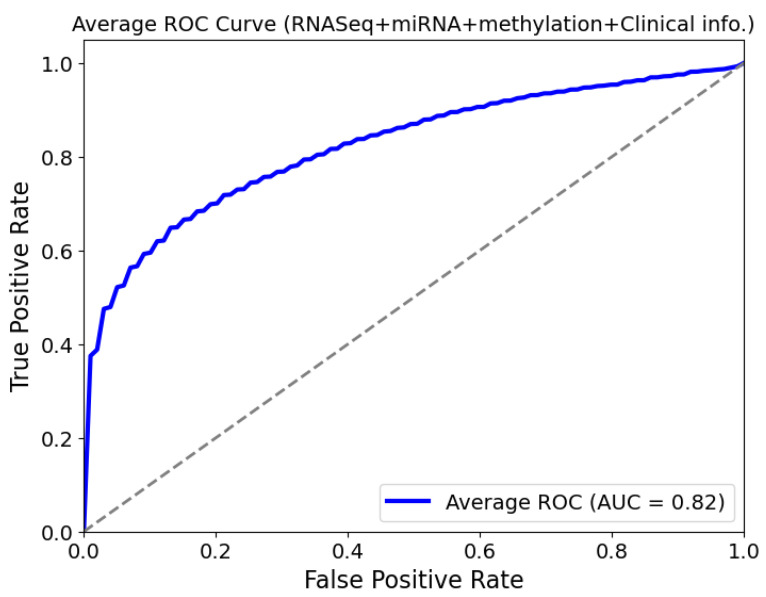
Average ROC curve for cancer survival prediction using mRNA, miRNA, methylation, and clinical information (AUC = 0.82).

Figure 5 shows the ROC curves for the NSCLC survival prediction using our GAT model based on the multi-omics data plus clinical information. It can be noted that our GAT model achieved an area under the curve (AUC) of 0.82. This value is better than that achieved by Yimeng Guo [18] (AUC = 0.71) and Suniy Zheng et al. [17] (AUC = 0.76). This means that our model is capable of predicting survival analysis well based on the multi-omics data and the clinical information by utilizing the correlation between the samples as a graph structure. Many researchers have determined that the area under the ROC curve (AUC) is equal to the C-index, which measures the model performance in survival analysis [32,33,34]. This agrees with our results, as our model’s C-index value is equal to the value of AUC (0.82), confirming the validity of our GAT model robustness in this context. Also, it is worth mentioning that our model achieves an accuracy of 0.75 in predicting NSCLC.

### 4.3. Kyoto Encyclopedia of Genes and Genomes (KEGG) Pathway Analysis

**Figure 6:**
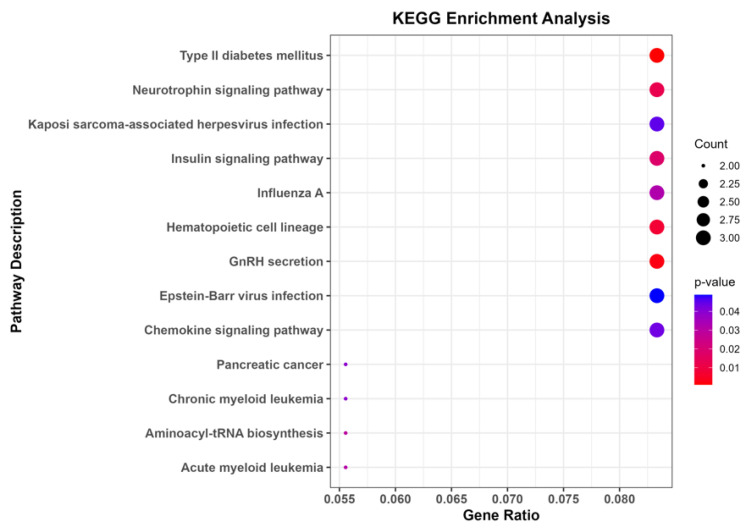
KEGG pathways analysis.

The features that had high attention weights in the GAT model were selected for further analysis. These features will have the highest biological relevance to the survival status of the patients and therefore indicate high confidence during the pathway analysis. The number of features that was used in the KEGG pathway analysis was 7425. During the process of pathway analysis, only 109 of these features could be converted to gene names using the *clusterProfiler* package version 4.12.2 in R. With the most recent gene annotation, the *clusterProfiler* package covers the functional properties of genomics data, both coding and non-coding, for thousands of species [35]. The KEGG analysis showed that the 109 genes were involved significantly in 13 enriched pathways with a *p*-value of less than 0.05. These 13 pathways included Type II diabetes mellitus, the neurotrophin signaling pathway, Kaposi sarcoma-associated herpesvirus infection, and pancreatic cancer, among others, as depicted in Figure 6. Although there is no direct lung cancer-specific pathway among the listed pathways, some of these pathways such as the chemokine signaling pathway and neurotrophin signaling pathway can be relevant to lung cancer. These pathways are involved in immune response, inflammation, and signal transduction, which are deemed to motivate cancer development, including lung cancer. The ratio on the *x*-axis in Figure 6 represents the percentage ratio of our selected genes to the genes in each pathway; the size of the dots in the figure represents the number of the genes that contribute to the pathway. The bar itself consists of several shades of gray, each corresponding to the path’s significance level; the darker the shade, the higher the *p*-value. These subsets provide essential information with which to understand the biological hallmarks in lung cancer pathophysiology. This identification of these pathways means that these genes can be crucial in the development stages of lung cancer and might be good subjects for therapeutic intervention. It also demonstrates how the GAT should be employed to identify biomarkers which, with an attention weight of 1, provide more robust and effective pathway analysis. Furthermore, there are other works that have connected some of the pathways that are presented in Figure 6 to lung cancer. For instance, pathways such as Type II diabetes mellitus [36,37], Influenza A [38,39], and Epstein–Barr virus infection [40,41] are found to be associated with lung cancer risk, and this supports our findings.

## 5. Discussion

Our findings confirm that integrating multi-omics data significantly enhances the performance of survival models for lung cancer patients. Using the technique of the graph attention network (GAT), our approach made it possible to utilize the relationships which are actually embedded in multi-omics data for improved cancer prognosis. The improvement in the predictivity of the mRNA data combined with miRNA data also underlines the synergism of both molecular levels. This integrative approach is a move away from the single-omics analyses, which are often limiting in the context of tumor biology since they do not consider the complexity of the system.

When performing the feature selection, the chi-square test helped to enhance the accuracy of the predictions of the survival as well as make the chosen set of features more relevant and easier to interpret. Thus, before training the GAT model, the necessity of using the SMOTE technique to balance the dataset in order to avoid the problem of class imbalance was proclaimed as one of the important goals when working with survival analysis, especially in dealing with censored data. This methodological consideration helped to make the model reliable and valid across different groups of patient populations.

Regarding the KEGG pathways, our GAT model identified key genes associated with critical biological functions and disease processes. For instance, the pathways, which the gene ontology classification borrowed, included Type II diabetes mellitus, Influenza A, and Epstein–Barr virus infection, to name but a few. These pathways have previously been linked with regard to cancer progression; they are involved in signaling, inflammation, and immune response; this is especially relevant in the case of lung cancer. The identification of these pathways not only affords the biological support for the selected features but can also offer potential drug intervention sites. From a survival prediction perspective, these pathways provide important information regarding how molecular mechanisms alter the prognosis of patients. For instance, diabetes mellitus pathways have been associated with poor survival in NSCLC patients [42] because the metabolic disturbances common in cancer patients can stimulate tumor progression [43]. In a similar manner, viral entry pathways, such as the Epstein–Barr virus and Influenza A, are known to alter immune responses that can either promote or inhibit tumor clearance based on the immune context of the patient [44]. These pathways may contribute to inflammation and immune suppression, which may be detrimental to NSCLC patients because the tumor may be able to escape immune surveillance.

From a clinical perspective, this integrative approach can have a positive impact on the management of patients by predicting more accurate prognostic models. Clinical professionals can gain a deeper understanding of the basic mechanisms behind the advancement of lung cancer by capturing numerous layers of molecular interactions. Among its potential benefits, it can result in the defining of the specific patient populations that may require more specialized treatment, which may otherwise prolong their survival. Also, utilizing the chi-square test for feature selection can pick the most important features that have a profound effect in NSCLC, making it easier for clinicians to focus on the most biologically relevant features.

The challenges that this study faced included the different scales of data and noise that are associated with omics data types, making data integration a challenge. Nevertheless, the significant promise of the GAT model was clear in the extent to which it accurately predicted the survival in patients, compared to other state-of-the-art methods. It is suggested that such studies should be pursued in the future in order to apply more detailed analyses to integrate other layers of omics data and even better methods of feature selection that would increase the model’s accuracy and clinically relevant predictive capabilities. Pathway analysis complements the use of the models in providing an understanding of the biological functions underlying the indices and, therefore, constitutes a step towards the incorporation of higher levels of abstraction which could be used to design specific therapies for lung cancer patients. The limitations of this study that must be acknowledged include the following: (1) The dataset is obtained from a public data repository (TCGA), which may not be representative of the broader real-world population of patients. (2) The SMOTE may introduce noise that affects the biological relevance of the data. Therefore, further work that uses larger and heterogeneous populations should be conducted. Also, a more sophisticated data-balancing technique can be utilized.

## 6. Conclusions

The major contribution of this work is the development of a new framework for the survival analysis of lung cancer based on multi-omics data by employing the graph attention network. When using a balanced set of samples and applying feature selection methods to the GAT model, outstanding predictive values were obtained and using both mRNA and miRNA data gave the highest accuracy. Our KEGG analysis of the selected significant features showed that they were implicated in pivotal biological processes underlying pathways such as Type II diabetes mellitus, Influenza A, and the Epstein–Barr virus infection, which are linked to lung cancer progression. These pathways support the biological significance of the features detected by the GAT model and contribute to the approval of its usefulness as a predictive biomarker. The results emphasize the importance of a multi-omics approach to improving several survival models of lung cancer patients. For the future studies, more samples should be collected, and more omics data should be added to the dataset to provide more evidence for the model, and further study of the GAT model should be conducted to check the best ways to interpret the model in order to provide it for clinical diagnosis. The directions proposed in this study are a substantial contribution to current and future developments in the utilization of high-dimensional biological data for clinical diagnosis and treatment, thus opening new horizons for future work and study in the analysis of cancer genomics.

## Figures and Tables

**Table 1:** The clinical information data.

Characteristics	Statistics	Missing
Total Number	624	
Gender		0
Male	370 (59.29%)	
Female	254 (40.71%)	
Age		12
Average (std)	66.19 (9.32)	
Age range	39–88	
Prior Malignancy		1 not reported
Yes	86 (13.80%)	
No	537 (86.20%)	
Synchronous malignancy		43 not reported
Yes	13 (2.24%)	
No	568 (97.76%)	
Prior Treatment		0
Yes	3 (0.48%)	
No	621 (99.52%)	
Primary diagnosis		0
Adenocarcinoma	348 (55.77%)	
Squamous cell carcinoma	276 (44.23%)	
Tumor stage		0
T1, T1a, T1b	209 (33.49%)	
T2, T2a, T2b	319 (51.12%)	
T3	78 (12.50%)	
T4	15 (2.40%)	
TX	3 (0.48%)	
Lymph node stage		1
N0	433 (69.50%)	
N1	127 (20.38%)	
N2	51 (8.19%)	
N3	11 (1.77%)	
NX	1 (0.16%)	
Metastasis stage		4
M0	428 (69.03%)	
M1, M1a, M1b	12 (1.93%)	
MX	180 (29.03%)	
Tissue organ		0
Upper lobe, lung	361 (57.85%)	
Lower lobe, lung	214 (34.29%)	
Middle lobe, lung	21 (3.37%)	
Lung, NOS	14 (2.24%)	
Overlapping lesion of lung	8 (1.28%)	
Main bronchus:	6 (0.96%)	
No. of pack-years smoked		154
Average (std)	47.09 (28.43)	

**Table 2:** Chi-square test results for significant feature selection across omics data.

Data Type	Number of Significant Features	Min *p*-Value	Max *p*-Value
mRNA Data	2945	3.7131 × 10^−7^	0.04998
miRNA Data	77	0.0002	0.0315
DNA Methylation Data	13,046	6.9051 × 10^−6^	0.0315

**Table 3:** C-index values for different combinations of omics data and clinical information.

Data Combination	Number of Features	Average C-Index
mRNA-miRNA-Meth-ClinicInfo	16,079	0.81693
mRNA-miRNA-Meth	16,068	0.81439
mRNA-miRNA	3022	0.86322
mRNA-Meth	15,991	0.81750
miRNA-Meth	13,123	0.79529
mRNA-ClinicInfo	2956	0.86056
miRNA-ClinicInfo	88	0.84738
Meth-ClinicInfo	13,057	0.79437
mRNA-miRNA-ClinicInfo	3033	0.86092
mRNA-Meth-ClinicInfo	16,002	0.82184
miRNA-Meth-ClinicInfo	13,134	0.79463
mRNA	2945	0.85676
miRNA	77	0.83605
Meth	13,046	0.79091
ClinicInfo	11	0.62480

**Table 4:** Comparative analysis of predictive models for non-small cell lung cancer.

Author	Method	Features	Accuracy	C-index
Our Method	Graph attention network	Multi-omics data + Clinical Information	0.75	0.82
Jacob G. Ellen [13]	autoencoders	Omics data + Clinical Information		(LUAD 0.69) (LUSC 0.62)
Zhang, J [14]	Nomogram model	Clinical Information		0.71
She Y et al. [15]	Deep learning based-algorithm	Clinical Information		0.74

## Data Availability

The used data and the model’s code will be available upon request.
